# Contextualizing the Price of Biosimilar Adalimumab Based on Historical Rebates for the Original Formulation of Branded Adalimumab

**DOI:** 10.1001/jamanetworkopen.2023.23398

**Published:** 2023-07-13

**Authors:** Sean R. Dickson, Nico Gabriel, Inmaculada Hernandez

**Affiliations:** 1 West Health Policy Center, Washington, DC; 2 Division of Clinical Pharmacy, Skaggs School of Pharmacy and Pharmaceutical Sciences, University of California, San Diego, La Jolla

## Abstract

This cross-sectional study compares recent list and net prices for Humira after rebates with announced prices of interchangeable biosimilar Humira formulations.

## Introduction

In 2023, branded adalimumab (Humira) saw the end of its 20-year market exclusivity. When launching Amjevita in January 2023, the first biosimilar adalimumab marketed, Amgen established 2 versions, one with a list price 55% below Humira and a second with a list price 5% below Humira, with presumably a 50% rebate.^1^ To our knowledge, despite the long-anticipated entry of biosimilar adalimumab, no study has quantified net prices faced by payers after rebates for Humira. This information contextualizes the prices announced for biosimilars and enables estimations of savings associated with their entry.

We estimate commercial (private market and Medicare Part D) and Medicaid net prices of Humira after accounting for rebates negotiated with pharmacy benefit managers (PBMs). We then compare recent list and net prices for Humira with announced biosimilar prices.

## Methods

This study was exempt from institutional review board approval as research without human participants, and data were deidentified. For 2013-2020, we estimated annual gross sales as the product of list price and units sold.^2,3^ We then estimated the difference between gross sales and company-reported net sales from SSR Health.^3^ From this difference, we subtracted government discounts (Medicaid, 340B, and coverage gap) and attributed the remaining amount to PBM rebates. We estimated Medicaid and 340B discounts under an established method, accounting for the Medicaid best price, as detailed in the eMethods in Supplement 1.^4,5^ Medicaid inflation rebates, which reimburse Medicaid for price increases above inflation, were separately estimated for original Humira and the citrate-free version introduced in 2018. Coverage gap discounts were estimated from the Medicare 5% sample. We assume both original and citrate-free Humira have the same net price after PBM rebates. This study followed the STROBE reporting guideline for cross-sectional studies.

## Results

**Figure 1.**
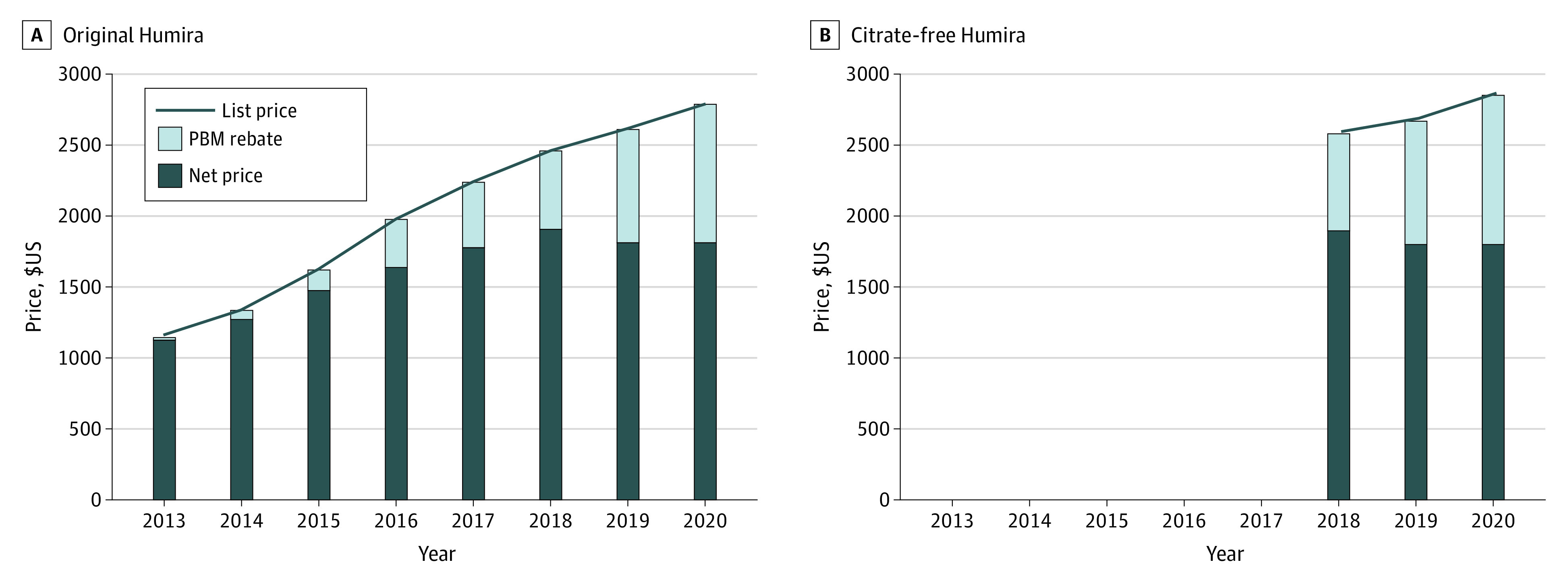
Trend in List Price, Rebates to Pharmacy Benefit Managers (PBMs), and Net Prices Faced by Commercial and Part D Plans of Humira, 2013-2019 Trends in nominal list price, rebates to PBMs, and resulting net prices faced by commercial and Part D plans for original (A) and citrate-free (B) Humira (branded adalimumab). Outcomes are expressed per 40 mg of Humira, which corresponds to a pen or syringe of original Humira or half a pen or syringe of citrate-free Humira. The bar components represent the proportion of the list price accounted for by rebates to PBMs vs net price. Net price is the same in both the original and citrate-free formulations; however, observed list price based on reimbursement varies slightly across the 2 products, resulting in different effective rebate values.

In 2013-2020, the list price for original Humira increased from $1153 to $2784 (141% increase) and PBM rebates increased from $28 (2.4% of list price) to $973 (34.9% of list price) (Figure 1A). Average net price for commercial and Part D plans increased from $1125 in 2013 to $1906 in 2018 (69% increase) and then decreased to $1812 in 2020 (65.1% of list price).

**Figure 2.**
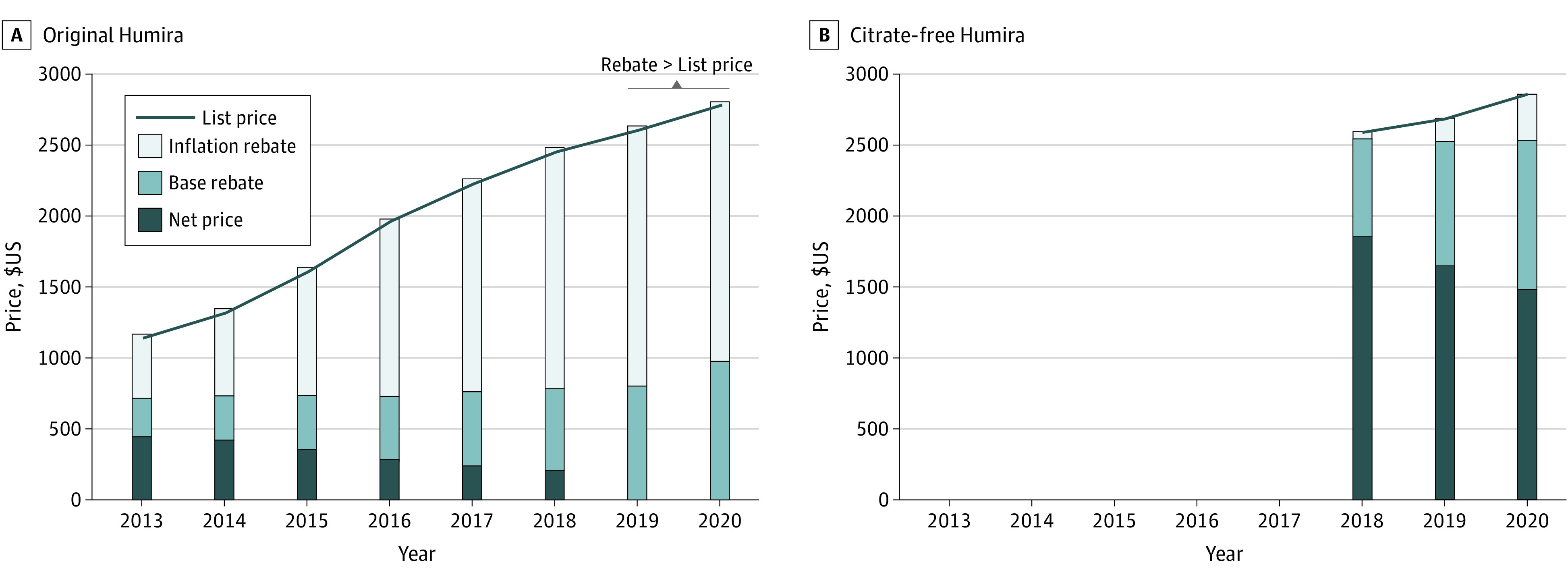
Trend in Medicaid Rebates and Net Prices of Humira, 2013-2019 Trends in nominal list price, Medicaid base rebates, inflation rebates, and resulting net prices faced by Medicaid for original (A) and citrate-free (B) Humira (branded adalimumab). Outcomes are expressed per 40 mg of Humira, which corresponds to a pen or syringe of original Humira or half a pen or syringe of citrate-free Humira. The bar components represent the proportion of the list price accounted for by the base rebate, the inflation rebate, and the net price. Net prices for original Humira are not represented in 2019-2020 because the combination of the base rebate and the inflation rebate triggered the Medicaid cap (where Medicare rebates exceed the list price and are capped at the list price of the drug).

From 2013 to 2018, the Medicaid base rebate equaled 23.1% of list price (Figure 2). After 2019, PBM rebates exceeded 23.1% of list price and therefore set best price. Medicaid inflation rebates for original Humira increased from 38.6% of list price in 2013 to 69.6% in 2019, when total rebates exceeded list price (Figure 2A). Medicaid net price for original Humira decreased from 38.3% of list price in 2013 to 8.3% in 2018 to 0% in 2019, when rebates exceeded list price. Inflation rebates were considerably lower for citrate-free Humira, resulting in a substantially higher Medicaid net price (Figure 2B).

## Discussion

Despite increasing rebates, the net price of Humira faced by commercial and Part D plans in 2020 was $1812, 3.5 times the launch price ($522). In 2023, the lowest-cost formulation of Amjevita had a list price of $1558, a 14% discount from the 2020 net price of Humira. Even at the 55% discount, Amjevita still costs more than double the launch price of Humira. These estimations are limited by only being able to compare the 2020 net price of Humira with current Amjevita pricing and by the lack of data to estimate discounts to federal purchasers, which are as a result included under PBM rebates.

While Amjevita is not interchangeable with Humira, interchangeable versions of Humira are anticipated to launch in the second half of 2023. These formulations may compete more directly on list price, marketing to pharmacies seeking to source lowest-cost product to generate a margin when reimbursed by an insurer at a predetermined amount for interchangeable formulations. However, the interchangeable biosimilars expected in 2023 will only be interchangeable with original Humira, but not the citrate-free version.^6^

## Notes

Supplement 1.
eMethods

eReferences

Supplement 2.
Data Sharing Statement
