# Supplementary material for: Contextualizing the Price of Biosimilar Adalimumab Based on Historical Rebates for the Original Formulation of Branded Adalimumab
Source: JAMA Netw Open. 2023 Jul 13;6(7):e2323398. doi: 10.1001/jamanetworkopen.2023.23398 (PMC10346129; doi:10.1001/jamanetworkopen.2023.23398)
Supplement: Supplement 1. — eMethods eReferences [file jamanetwopen-e2323398-s001.pdf]

Supplemental Online Content

Dickson SR, Gabriel N, Hernandez I. Contextualizing the price of biosimilar adalimumab based on historical rebates for the original formulation of branded adalimumab. *JAMA Netw Open.* 2023;6(7):e2323398. doi:10.1001/jamanetworkopen.2023.23398

eMethods

eReferences

.This supplemental material has been provided by the authors to give readers additional information about their work.

© 2023 Dickson SR et al. *JAMA Network Open*.

eMethods

We use peer-reviewed methodology to estimate net prices for Humira.[1,2] In brief, we estimated total discounts as the difference between gross sales and net sales. We estimates annual gross sales as the product of list price and units sold, from IQVIA National Sales Perspective[3] and SSR Health,[4] which in turns obtains estimates of units from Symphony Health.[5] These are commercial databases that estimate the number of units sold for a drug across the US. From the total discount amount estimated, we subtract government discounts (Medicaid, 340B, and coverage gap), and attribute the remaining amount to PBM rebates. Medicaid and 340B discounts are estimated as explained below, while coverage gap discounts are estimated using claims data from a 5% random sample of Medicare beneficiaries.

Our methodology for the estimation of Medicaid discounts and pharmacy benefit manager discounts relies on the relationship between rebates and Medicaid discounts established by the Best price provision.[6]

Medicaid discounts= number of Medicaid units * Medicaid discount per unit

Number of Medicaid units were obtained from the Centers for Medicare and Medicaid Services dashboard.

Medicaid discount per unit=base rebate + inflation penalty

- The inflation penalty was calculated each year as the difference between the list price and the inflation-adjusted launch price, following a published method.[7] We separately estimated the inflation penalty for original Humira and the citrate-free version introduced in 2018.

  Launch price data is available at the national drug code level and our analyses were conducted at the product level. To estimate an inflation-adjusted launch price at the product level, we calculated the inflation-adjusted launch price for each national drug code every year, and weighted by the relative utilization of each national drug code every year.
- The base rebate is calculated as the greater of 23% discount or the Best Price offered to any purchaser. To estimate the base rebate, in the first iteration of our algorithm, we assumed that the rebate set Best Price. Therefore, we subtracted the inflation Medicaid and 340B inflation penalty discounts from the total discounts figure.

  Total discounts- Medicaid inflation penalty- 340B inflation penalty-coverage gap discounts=**x**
  We amortize the remaining discount **x** amount across the sum of group health plans, Part D, Medicaid, and 340B program units to generate the estimated rebate per unit. Then, the total rebate amount is estimated as the product of the rebate per unit and commercial units (group health insurance and Medicare Part D units).
  If the estimated rebate ≥23.1% of list price, then the assumption that the rebate set Best Price holds, as it was the case in 2019 and 2020.
  If the estimated rebate < 23.1% of list price, then the rebate does not trigger the Best Price provision. This was the case in 2013-2018. For those years, we re-

© 2023 Dickson SR et al. *JAMA Network Open*.

estimate Medicaid and 340B discounts as the sum of 23.1% of list price and inflation penalty. We subtract these re-calculated discounts to the Medicaid program and 340B discounts from the total discounts figure. The resulting estimate **y** represents rebates.

Total discounts- Medicaid units *(23% of list price + inflation penalty)- 340B units* (23% of list price + inflation penalty)-coverage gap discounts=**y**

Medicaid and 340B discounts are capped at 100% of the Average Manufacturer Price, in other words, the sum of the base discount and the inflation penalty cannot exceed the invoice price. We checked whether the sum of the estimated base rebate and inflation penalty exceeded the list price. This was the case of the original formulation of Humira in 2019-2020. Then, in 2019-2020, we recalculated the total rebate amount as follows, where **z** represents rebates:

Total discounts- Medicaid units*list price-340B units * list price-coverage gap discounts=**z**

© 2023 Dickson SR et al. *JAMA Network Open*.

eReferences

1. Dickson S, Gabriel N, Gellad WF, Hernandez I. Assessment of Voluntary and Mandatory Discounts in the Gross-to-Net Bubble for Leading Insulin Products, 2012-2019. *JAMA Netw Open In Press*.

2. Dickson S, Gabriel N, Gellad WF, Hernandez I. Estimated Changes in Insulin Prices and Discounts following Entry of New Insulin Products, 2012-2019. *JAMA Health Forum In Press*.

3. US National Sales Perspectives TM January 2007-December 2022, IQVIA Inc.. All Rights Reserved.

4. SSR Health. Accessed September 14, 2022. https://www.ssrhealth.com/dataset/

5. Symphony Health Data Overview. Accessed September 14, 2022. https://symphonyhealth.com/what-we-do/view-health-data

6. Dickson S, Gabriel N, Hernandez I. Estimated Changes in Price Discounts for Tenofovir-Inclusive HIV Treatments Following Introduction of Tenofovir Alafenamide. *AIDS 2022 doi: 101097/QAD0000000000003401*.

7. Dickson S, Gabriel N, Gellad W, Hernandez I. Reduction in Medicaid rebates paid by pharmaceutical manufacturers for outpatient injected, inhaled, infused, implanted, or instilled drugs: The 5i loophole. *J Health Polit Policy Law*. Published online July 14, 2022. doi:10.1215/03616878-10041219

© 2023 Dickson SR et al. *JAMA Network Open*.
