# Supplementary material for: Contextualizing the Price of Biosimilar Adalimumab Based on Historical Rebates for the Original Formulation of Branded Adalimumab
Source: JAMA Netw Open. 2023 Jul 13;6(7):e2323398. doi: 10.1001/jamanetworkopen.2023.23398 (PMC10346129; doi:10.1001/jamanetworkopen.2023.23398)
Supplement: Supplement 2. — Data Sharing Statement [file jamanetwopen-e2323398-s002.pdf]

# Data Sharing Statement

Dickson. Contextualizing the Price of Biosimilar Adalimumab Based on Historical Rebates for the Original Formulation of Branded Adalimumab. *JAMA Netw Open*. Published July 13, 2023. doi:10.1001/jamanetworkopen.2023.23398

**Data**
**Data available:** No

**Additional Information**
**Explanation for why data not available:** Data were obtained under a data user agreement that prevents data sharing
